# RETRACTION: The PI3K/mTOR Dual Inhibitor NVP‐BEZ235 Stimulates Mutant p53 Degradation to Exert Anti‐Tumor Effects on Triple‐Negative Breast Cancer Cells

**DOI:** 10.1002/2211-5463.70196

**Published:** 2026-01-20

## Abstract

**RETRACTION**: J. Cai, J. Xia, J. Zou, Q. Wang, Q. Ma, R. Sun, H. Liao, L. Xu, D. Wang, and X. Guo, “The PI3K/mTOR Dual Inhibitor NVP‐BEZ235 Stimulates Mutant p53 Degradation to Exert Anti‐Tumor Effects on Triple‐Negative Breast Cancer Cells,” *FEBS Open Bio* 10, no. 4 (2020): 535–545, https://doi.org/10.1002/2211‐5463.12806.

The above article, published online on 06 February 2020 in Wiley Online Library (wileyonlinelibrary.com), has been retracted by agreement between the journal Editor‐in‐Chief, Miguel De la Rosa; the Federation of European Biochemical Societies; and John Wiley & Sons Ltd. The retraction has been agreed upon following an investigation into concerns raised by a third party. The investigation found multiple duplications of western blot bands among Figures 2A, 2B, 3A, 4A, 4B, 4C, 5A, and 5C. An overlap was also detected between the MDA‐MB‐468 0h CON image and the MDA‐MB‐468 0 h BEZ235 image shown in Figure 1B. The authors did not respond to address the concerns raised. Given the extent of the identified issues, the editors have lost confidence in the data presented and consider the conclusions of this manuscript substantially compromised. As a result, the Editor‐in‐Chief, FEBS Press, and John Wiley and Sons Ltd. have determined that a retraction is necessary. The authors were informed of the decision to retract but did not respond to requests for comment.

**RETRACTION**: J. Cai, J. Xia, J. Zou, Q. Wang, Q. Ma, R. Sun, H. Liao, L. Xu, D. Wang, and X. Guo, “The PI3K/mTOR Dual Inhibitor NVP‐BEZ235 Stimulates Mutant p53 Degradation to Exert Anti‐Tumor Effects on Triple‐Negative Breast Cancer Cells,” *FEBS Open Bio* 10, no. 4 (2020): 535–545, https://doi.org/10.1002/2211‐5463.12806.

The above article, published online on 06 February 2020 in Wiley Online Library (wileyonlinelibrary.com), has been retracted by agreement between the journal Editor‐in‐Chief, Miguel De la Rosa; the Federation of European Biochemical Societies; and John Wiley & Sons Ltd. The retraction has been agreed upon following an investigation into concerns raised by a third party. The investigation found multiple duplications of western blot bands among Figures 2A, 2B, 3A, 4A, 4B, 4C, 5A, and 5C. An overlap was also detected between the MDA‐MB‐468 0h CON image and the MDA‐MB‐468 0 h BEZ235 image shown in Figure 1B. The authors did not respond to address the concerns raised. Given the extent of the identified issues, the editors have lost confidence in the data presented and consider the conclusions of this manuscript substantially compromised. As a result, the Editor‐in‐Chief, FEBS Press, and John Wiley and Sons Ltd. have determined that a retraction is necessary. The authors were informed of the decision to retract but did not respond to requests for comment.

